# Histone deacetylase inhibition enhances the therapeutic effects of methotrexate on primary central nervous system lymphoma

**DOI:** 10.1093/noajnl/vdaa084

**Published:** 2020-07-03

**Authors:** Kenji Fujimoto, Naoki Shinojima, Mitsuhiro Hayashi, Tomoyuki Nakano, Koichi Ichimura, Akitake Mukasa

**Affiliations:** 1 Department of Neurosurgery, Kumamoto University Hospital, Kumamoto, Japan; 2 Division of Brain Tumor Translational Research, National Cancer Center Research Institute, Tokyo, Japan; 3 Division of Molecular Pharmacology, National Cancer Center Research Institute, Tokyo, Japan; 4 Department of Neurosurgery, Tokyo Medical and Dental University, Tokyo, Japan

**Keywords:** histone deacetylase inhibitor, leucovorin, methotrexate, polyglutamylation, primary central nervous system lymphoma

## Abstract

**Background:**

Polyglutamylation is a reversible protein modification that commonly occurs in tumor cells. Methotrexate (MTX) in tumor cells is polyglutamylated and strongly binds to dihydrofolate reductase (DHFR) without competitive inhibition by leucovorin. Therefore, tumor cells with high polyglutamylation levels are supposed to be selectively killed, whereas normal cells with lower polyglutamylation are rescued by leucovorin. This study investigated the combined effects of MTX plus histone deacetylase inhibitors (HDACIs), which upregulate MTX polyglutamylation, in primary central nervous system lymphoma (PCNSL).

**Methods:**

We evaluated cell viability after MTX treatment and leucovorin rescue and compared the expression of folylpolyglutamate synthetase (FPGS), γ-glutamyl hydrolase (GGH), and DHFR in 2 human PCNSL-derived cell lines (HKBML and TK) and a human Burkitt lymphoma cell line (TL-1). Combination treatments were created using 4 HDACIs: panobinostat, vorinostat, sodium butyrate, and valproic acid. The expression of DHFR was examined as well as ratios of FPGS/GGH expression. The combined effects of MTX plus HDACIs were evaluated using a cell viability assay, mass spectroscopy imaging, and subcutaneous and intracranial xenograft models.

**Results:**

HDACIs upregulated the ratio of FPGS/GGH expression resulting in increased polyglutamylation of MTX, but also downregulated expression of the target molecule of MTX: DHFR. The combination of MTX and vorinostat decreased cell viability in vitro (*P* < .05) and tumor volumes in a subcutaneous model (*P* < .0001), and prolonged survival in an intracranial model (*P* < .01), relative to controls.

**Conclusion:**

HDACIs enhanced the therapeutic effect of MTX through increased polyglutamylation of MTX and concomitant downregulation of DHFR expression.

**Key Points**

- Primary central nervous system lymphoma was treated using methotrexate + leucovorin (LV).
- The response to MTX + LV was dependent on the extent of MTX polyglutamylation.
- Increasing MTX polyglutamylation and decreasing DHFR expression are more efficacious.

**Importance of the Study**

The standard treatment for primary central nervous system lymphoma (PCNSL) is high-dose methotrexate (MTX)-based chemoradiotherapy with leucovorin rescue. After MTX is transported into tumor cells, it is polyglutamylated and retained inside the cell, where it strongly binds to dihydrofolate reductase (DHFR). Polyglutamylated MTX is not subject to competitive inhibition by leucovorin, which results in long-lasting inhibition of DHFR. Therefore, tumor cells with high polyglutamylation levels are selectively killed, whereas normal cells with lower polyglutamylation are rescued by leucovorin. We found that histone deacetylase inhibitors induce downregulation of DHFR expression and increase the polyglutamylation of MTX, resulting in enhancing the antitumor effect of MTX in in vitro and in vivo assays in PCNSL. This suggests that HDACIs might enhance the antitumor effect of MTX, which needs to be tested in preclinical research on patients with MTX-resistant intractable PCNSL.

Primary central nervous system lymphoma (PCNSL) is an extranodal non-Hodgkin lymphoma that arises in, and is confined to, the CNS. It has a low tendency for systemic dissemination and a relatively aggressive course, which is distinct from systemic lymphoma.^1–3^ The standard treatment for PCNSL is high-dose methotrexate (HD-MTX)-based chemoradiotherapy with leucovorin (LV) rescue.^4,5^

Folate is necessary for the de novo biosynthesis of purines and thymidylate, so it is an essential factor for DNA synthesis.^6^ The monoglutamate form of folate is the only one circulating in the blood and can be transported across the cell membrane. Once folate is taken up into cells, intracellular folate exists primarily as polyglutamate. Intracellular folate is converted to polyglutamate by folylpolyglutamate synthetase (FPGS), while γ-glutamyl hydrolase (GGH) removes the terminal glutamates. Polyglutamylated folates are better retained in cells and are better substrates than monoglutamates for intracellular folate-dependent enzymes.^6^

MTX is a classical folate antagonist. Its antitumor mechanism is attributed to inhibition of dihydrofolate reductase (DHFR)-mediated regeneration of tetrahydrofolates from dihydrofolates. As with folate, MTX is retained in tumor and normal cells by FPGS-induced polyglutamylation and is exported from cells after hydrolysis to monoglutamates by GGH.^7,8^

Long-chain polyglutamylated MTX (MTX-PG) inhibits not only DHFR but also other important folate pathway enzymes in the thymidylate and purine biosynthetic pathways, such as thymidylate synthase (TS), to a greater extent than MTX monoglutamate forms.^9^ Therefore, concomitant inhibition of DHFR and TS leads to enhanced impairment of DNA synthesis and subsequent cell-cycle arrest, enhancing the cytotoxic effect of MTX-PG.

Leucovorin is a reduced form of folate, which is converted to tetrahydrofolate, bypassing the inhibition of DHFR by MTX. LV also competes for binding with antifolates as a reduced folate carrier; when converted to tetrahydrofolate, it competes with antifolates for polyglutamylation.^10^

MTX-PG can strongly bind to DHFR and is minimally subject to competition by LV, which results in long-lasting inhibition of DHFR.^11–13^ Unlike normal cells, tumor cells exhibit frequent occurrence of polyglutamylation.^14^ Therefore, MTX treatment can selectively kill cancer cells with high levels of polyglutamylation, whereas normal cells with lower levels of polyglutamylation are rescued by LV.^15^

In our previous study, we found that the response to HD-MTX therapy with LV rescue was better in patients with PCNSL who had high polyglutamylation levels.^16^ In addition, we found that sodium butyrate (NaBu), which is a pan-histone deacetylase inhibitor (HDACI), upregulated the FPGS expression. Therefore, we hypothesized that the combination of MTX plus HDACIs might be more effective for treating PCNSL, via induction of MTX polyglutamylation, relative to conventional HD-MTX-based chemotherapy alone. The present study investigated the response of PCNSL to MTX plus HDACI, using in vitro and in vivo models.

## Materials and Methods

### Cell Lines and Cell Culture

We purchased a human PCNSL-derived cell line (HKBML) and a Burkitt lymphoma cell line (TL-1) from the RIKEN BioResource Center. We purchased a human PCNSL-derived cell line (TK) from the JCRB Cell Bank. The HKBML cells were maintained in Ham’s F-12 medium (Wako) supplemented with 15% fetal bovine serum (FBS). The TL-1 and TK cells were maintained in RPMI 1640 medium (Wako) supplemented with 10% FBS. All media were supplemented with 1% penicillin–streptomycin. All cultures were maintained at 37°C in a humidified atmosphere with 5% CO_2_.

### Chemicals

The MTX was purchased from Wako. For the mass spectrometry (MS), unlabeled polyglutamylated MTX standards (MTX-PG2 to MTX-PG7) were purchased from Schircks Laboratories, as conventional MTX is in the monoglutamate form. Panobinostat, vorinostat, sodium butyrate (NaBu), and valproic acid (VPA) were purchased from Schircks Laboratories. The MTX was dissolved in 0.05 M Na_2_CO_3_ and the pH was adjusted to 7.0 before use. The panobinostat, vorinostat, VPA, and NaBu were dissolved in DMSO for in vitro use. The vorinostat was dissolved in 2% DMSO + 30% PEG300 + 5% Tween80 + ddH_2_O for use in the xenograft mouse models.

### Cell Viability Assay

Lymphoma cells were seeded in 96-well plate at 10 000 cells per well. Six hours post-seeding, MTX or HDACIs cells were diluted and added to each well in 0.1% 0.05 M Na_2_CO_3_ or 0.1% DMSO final concentration, respectively. Vehicle control was 0.1% 0.05 M Na_2_CO_3_ for MTX or 0.1% DMSO for HDACIs. After 72 h, cell viability was measured by CellTiter-Glo (Promega) and the IC_50_ values were determined.

To evaluate the effect of MTX treatment and LV rescue, cells were treated using MTX (100 nM) for 24 h, which was followed by rescue using LV (Pfizer Japan Inc.). Cell viability assays were performed 48 h later. We defined the EC_50_ value as the LV concentration that recovered 50% cell viability relative to the control. To evaluate the combination of MTX and vorinostat, cells were exposed to MTX for 24 h, washed twice with cold phosphate-buffered saline (PBS), and treated with vorinostat for 24 h. The cells were then washed twice with cold PBS, resuspended in drug-free medium, and the cell viability assay was performed 48 h later.

### Western Blotting

Western blotting was performed as previously described.^17^ When each molecule was detected by use of the following primary antibodies, the same immunoblotting membrane was used for comparison of lanes adjacent to one another after correction by internal control. The primary antibodies were rabbit polyclonal anti-FPGS (1:1000; Spring Bioscience), rabbit monoclonal anti-γ-glutamyl hydrolase (GGH; 1:500; Abcam), rabbit monoclonal anti-DHFR (1:10 000; Abcam), and mouse monoclonal anti-α-tubulin (1:5000; Sigma-Aldrich). The densitometry of blots was analyzed using Image J software.^18^

### Matrix-Assisted Laser Desorption/Ionization Mass Spectrometry Imaging

The TL-1 cells were exposed to control medium or vorinostat-containing medium for 72 h, washed twice with cold PBS, and treated with MTX for 24 h. After being washed twice with cold PBS, the cell pellets were collected and stored at –80°C until the subsequent analysis. The matrix-assisted laser desorption/ionization mass spectrometry imaging (MALDI-MS imaging) methods are described in detail in Supplementary Methods. The frozen cells were placed on a glass slide (PRO-11, Matsunami Glass) and coated with α-cyano-4-hydroxycinnamic acid (CHCA, #476870; Sigma-Aldrich) using an automated SMALDI-Prep sprayer (TransMIT). The molecules in the TL-1 cells were analyzed using a MALDI imaging ion source (AP-SMALDI-10, TransMIT) coupled to an orbitrap mass spectrometer (Q Exactive; Thermo Fisher Scientific). The resulting data were processed using Mirion software (v3.2.64.18, TransMIT) as previously described.^19^ The relative amount of MTX-PG was then calculated based on correction using the number of detected pixels with phospholipids.

### Efficacy in a Xenograft Mouse Model

All animal experiments were approved by the Animal Experimental Committee of the National Cancer Center and were performed in accordance with the Guidelines for Animal Experiments of the National Cancer Center. These standards are aligned with the ethical guidelines for animal experiments in Japan.

We used mouse subcutaneous and brain xenograft models for the in vivo evaluation of the combination of MTX plus vorinostat. In the subcutaneous xenograft model, we injected TL-1 cells into 6-week-old male SCID Beige mice (Charles River). The cells were subcutaneously injected into the flank region to deliver 3.0 × 10^6^ cells in 100 μL of PBS. Fifteen days later, the mice were randomly divided into 4 groups: MTX alone (50 mg/kg), vorinostat alone (50 mg/kg), MTX (50 mg/kg) plus vorinostat (50 mg/kg), or vehicle control. MTX was dissolved in 0.05 M Na_2_CO_3_ diluted to 50% with saline. Vorinostat was dissolved in 2% DMSO + 30% PEG300 + 5% Tween80 + ddH_2_O. These treatments were administered intraperitoneally at days 15, 18, and 21. The tumor volume was measured 3 times per week using calipers: tumor volume (mm^3^) = (length × width^2^) / 2.

For the intracranial xenograft model, we injected 2.0 × 10^5^ TK cells in 2 μL of PBS into the right cerebral hemispheres of 6-week-old male BALB/c-nu/nu mice (Charles River) via a Hamilton syringe and stereotactic micro-injector (Narishige). Four days later, the mice were randomly divided into 4 groups: MTX alone (50 mg/kg), vorinostat alone (50 mg/kg), MTX (50 mg/kg) plus vorinostat (50 mg/kg), or a vehicle control. These treatments were administered intraperitoneally at days 4, 6, 8, and 10. The health status of each mouse was checked daily for sacrifice criteria. The sacrifice criteria were the loss of 20% body weight, ataxia, apathy, or paralysis. Mice fulfilling any one of these criteria were euthanized promptly.

### Immunohistochemical Staining

Immunostaining was performed using formalin-fixed paraffin-embedded tumor specimens and a rabbit monoclonal anti-CD20 antibody (1:250; Abcam), as previously described.^17^

### Statistical Analyses

Parametric analyses were performed using Student’s *t* test, based on the mean ± standard deviation. Survival analyses were performed using the Kaplan–Meier method and the log-rank test. Differences were considered statistically significant at *P* values of less than .05. All analyses were performed using IBM SPSS software (version 19; IBM Corp.).

## Results

### Effects of MTX Treatment and LV Rescue Were Related to Polyglutamylation in Lymphoma Cell Lines

**Figure 1.**
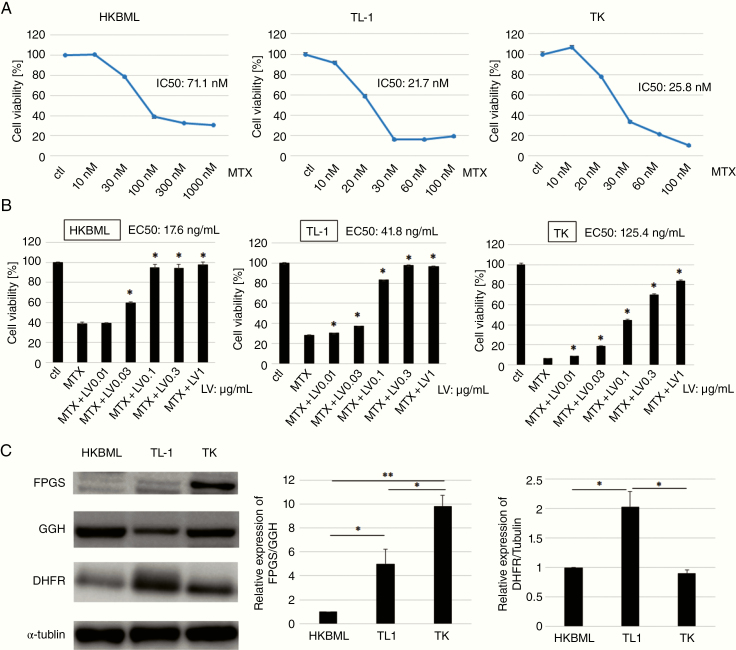
Effects of methotrexate (MTX) treatment and leucovorin (LV) rescue were related to the degree of polyglutamylation in lymphoma cell lines. (A) Viability assays using cell lines after incubation with MTX (CellTiter-Glo). Each cell line was analyzed after 72 h of incubation. (b) Cells were treated with MTX for 24 h, followed by the addition of LV. Cell viability was assessed 48 h later. We defined EC_50_ as the concentration of LV that recovered 50% cell viability. Data are shown as mean value ± SD from 3 independent experiments. *P < .01, compared with MTX. (C) Immunoblotting for folypolyglutamate synthetase (FPGS), γ-glutamyl hydrolase (GGH), and dihydrofolate reductase (DHFR) in the different cell lines. The internal control was α-tubulin. The relative expression of FPGS/GGH represents the ratio of FPGS/α-tubulin and GGH/α-tubulin calculated by densitometry. The FPGS/GGH ratio of HKBML is adjusted to 1. Data are shown as mean value ± SD from 3 independent experiments; *P < .05, **P < .01.

The cytotoxic effects of MTX on HKBML, TL-1, and TK cells were examined using the in vitro CellTiter-Glo assay. The IC_50_ values for MTX were 71.1 nM for HKBML cells, 21.7 nM for TL-1 cells, and 25.8 nM for TK cells (Figure 1A). When we used 100 nM of MTX, the cytotoxic effects reached almost maximum in all cell lines. There were a few differences in the cell viability between 100 and 1000 nM of MTX usage (data not shown). Cell viabilities of HKBML, TL-1, and TK cells in the maximal cytotoxic effects of MTX were about 30%, 20%, and 5%, respectively. Over 90% cytotoxicity was reached only in TK cells.

The EC_50_ was defined as the LV concentration to recover 50% cell viability, which was found to be 17.6 ng/mL for HKBML cells, 41.8 ng/mL for TL-1 cells, and 125.4 ng/mL for TK cells (Figure 1B). These results indicated that HKBML cells were more easily rescued by LV, relative to the other cell lines.

We compared the expressions of FPGS, GGH, and DHFR among all cell lines using Western blotting (Figure 1C). The results revealed that FPGS expression was highest in TK cells and that GGH expression was highest in HKBML cells. The expression level of DHFR was highest in TL-1 cells and was not different between HKBML and TK cells.

The FPGS/GGH ratio was highest in TK cells and lowest in HKBML cells, which was consistent with EC_50_ values for LV. Therefore, the response to MTX and LV rescue appears to be associated with the FPGS/GGH ratio, which nearly reflected the extent of MTX polyglutamylation.

### Effect of HDACIs on Lymphoma Cell Lines

**Figure 2.**
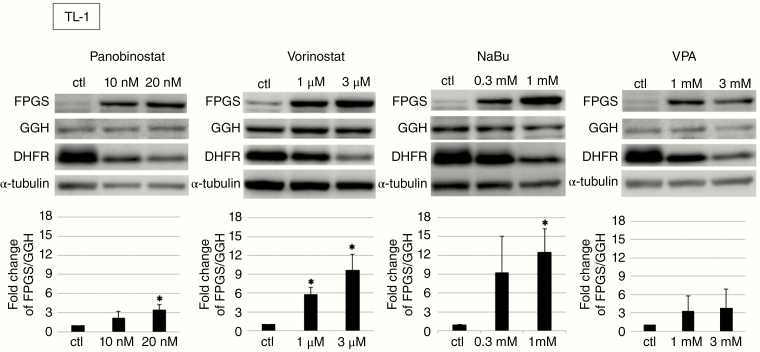
Changes in expression of folypolyglutamate synthetase (FPGS), γ-glutamyl hydrolase (GGH), and dihydrofolate reductase (DHFR) in TL-1 cells treated by histone deacetylase inhibitors (HDACIs) for 72 h. The relative expression of FPGS/GGH represents the ratio of FPGS/α-tubulin and GGH/α-tubulin calculated by densitometry. The FPGS/GGH ratio of TL-1 control is adjusted to 1. Data are shown as mean value ± SD from 3 independent experiments, *P < .05.

We examined the effects of HDACIs on lymphoma cell lines. In addition to NaBu, we used panobinostat, vorinostat, and VPA because these drugs are approved by the FDA for other diseases. The IC_50_ values for each drug were evaluated in the lymphoma cell lines (Table 1; Supplementary Figure S1). As the IC_50_ values for panobinostat and vorinostat were lower than the values for NaBu and VPA, panobinostat and vorinostat were more effective drugs than NaBu and VPA. We also evaluated whether HDACI treatment altered the expressions of FPGS, GGH, and DHFR (Figure 2; Supplementary Figure S2). Interestingly, all HDACIs not only upregulated the ratio of FPGS/GGH, which is supposed to induce increased polyglutamylation, but also downregulated the expression level of DHFR. These results implied that HDACIs might enhance the efficacy of MTX treatment.

**Table 1.** The IC_50_ Values for the Histone Deacetylase Inhibitors in Lymphoma Cell Lines

	Panobinostat	Vorinostat	NaBu	Valproic acid
HKBML	12.2 nM	1.5 μM	712 μM	1.3 mM
TL-1	4.6 nM	278 nM	617 μM	881 μM
TK	11.6 Nm	1.1 μM	1.5 mM	1.6 mM

NaBu, sodium butyrate.

### Combined Effect of MTX and Vorinostat

**Figure 3.**
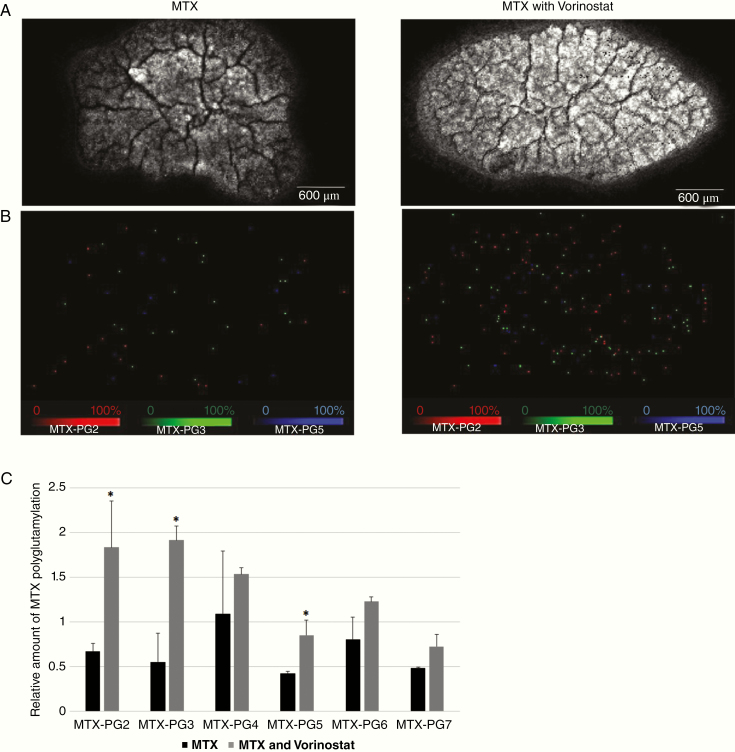
Molecular imaging of the distribution of methotrexate (MTX) polyglutamates 2–7 in TL-1 cells treated using vorinostat or vehicle followed by MTX. (A) Imaging of phospholipid (PC34:1), the surrogating cell component. (B) Distributions of MTX polyglutamates 2, 3, and 5. In the MALDI-MS imaging software, we could only display up to 3 channels at a time. Therefore, we used MTX-PG 2, 3, and 5 as representatives for figures because there were statistically significant differences in expression of MTX-PG 2, 3, and 5 between MTX and MTX plus vorinostat. (C) Relative amounts of MTX polyglutamates 2–7 are calculated by correcting the numbers of each detected pixel with phospholipid PC (34:1) in TL-1 cells, with or without vorinostat treatment. Error bars indicate standard deviation. *P < .05, compared with MTX.

For further exploration in patients with PCNSL, we evaluated whether adding vorinostat to MTX treatment was effective, since vorinostat passes through the blood–brain barrier.^20–22^ We used TL-1 cells to evaluate the intracellular accumulation of MTX-PGs using MALDI-MS imaging due to the higher upregulation of FPGS/GGH induced by vorinostat. The TL-1 cells were treated using MTX after pretreatment using the control solution or vorinostat, and the intracellular accumulation of MTX polyglutamates (MTX-PG2 to MTX-PG7) was evaluated using MALDI-MS imaging. Relative to cells treated using MTX alone, cells treated using vorinostat generally had higher levels of all MTX-PGs, with the statistical significance in MTX-PG2, 3, and 5 (*P* < .05; Figure 3A–C). These results confirmed that vorinostat caused the intracellular accumulation of long-chain MTX polyglutamates through the upregulation of FPGS, which should theoretically enhance the therapeutic effect of MTX thorough the long-lasting retention of MTX-PGs in tumor cells.

**Figure 4.**
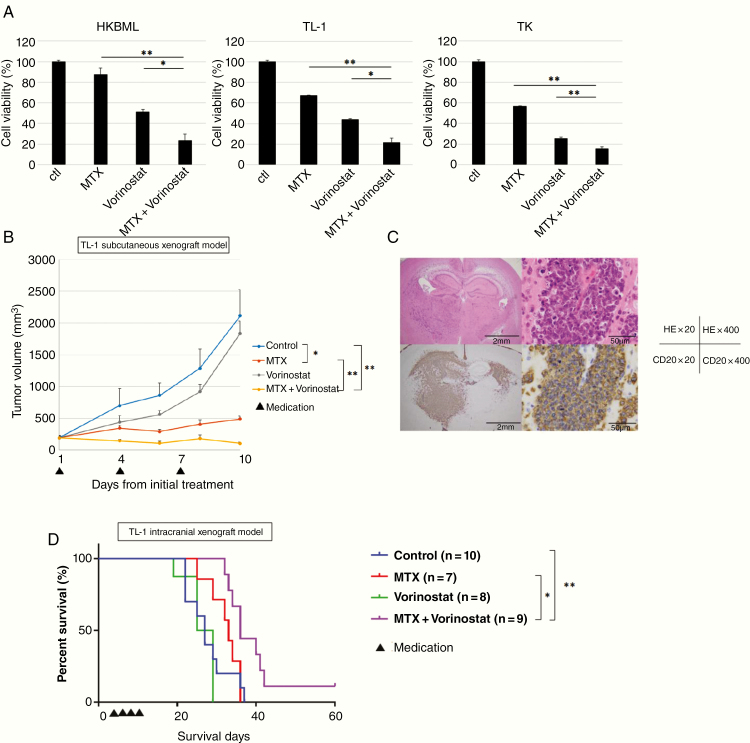
Effects of the methotrexate (MTX) plus vorinostat combination on lymphoma cell lines in vitro and in mouse xenograft models. (A) Cells were sequentially treated using MTX (24 h) and vorinostat (24 h), and cell viability was determined after 48 h of incubation in a drug-free medium. Error bars represent standard deviations. The viability of cells treated using MTX + vorinostat was compared to that of cells treated using MTX or vorinostat alone; *P < .05, **P < .01. (B) Effects of MTX and vorinostat in a mouse subcutaneous tumor model. Mice with TL-1 subcutaneous tumors received an intraperitoneal injection of vehicle, MTX (50 mg/kg), vorinostat (50 mg/kg), or MTX (50 mg/kg) + vorinostat (50 mg/kg) on days 15, 18, and 21. Error bars indicate standard deviation; *n* = 5 for each. *P < .0005, **P < .0001. (C) Xenograft tumor sections derived from the intracranial xenograft mouse model were stained using (Upper) hematoxylin and eosin or (Bottom) anti-CD20 to identify B-cell lymphomas. Scale bar, 2 mm (×20), 50 μm (×400). (D) Kaplan–Meier survival curves for mice harboring the TK intracranial xenografts after treatment using vehicle, MTX (50 mg/kg), vorinostat (50 mg/kg), or MTX (50 mg/kg) + vorinostat (50 mg/kg) The log-rank test was used for the statistical analysis. *P < .05, **P < .01.

Based on these findings, we tested the combination of MTX plus vorinostat on PCNSL cells in vitro. The cell viability assay revealed that this combination significantly decreased the viability of all cell lines, relative to MTX alone (Figure 4A). We also evaluated the combination of MTX and vorinostat in an in vivo model of PCNSL, using MTX at a dose of 50 mg/kg based on its known toxicity. The intracranial xenograft mouse model was created using TK cells and the mice were randomized into 4 groups: control, MTX alone, MTX with LV rescue at 6 h, and MTX with LV rescue at 24 h. Using the Kaplan–Meier method, we failed to detect significant intergroup differences in body weight or survival time (Supplementary Figure S3A and B). Given the lack of significant differences, we evaluated the effect of a combination of MTX plus vorinostat without LV rescue to determine whether this combination could induce MTX polyglutamylation.

To evaluate the combined effect of MTX plus vorinostat in the subcutaneous xenograft model, we used TL-1 cells due to the higher upregulation of FPGS/GGH induced by vorinostat in vitro study. We subcutaneously injected TL-1 cells into the flank regions of 6-week-old female SCID Beige mice, which were then randomized into 4 groups: vehicle control, MTX alone, vorinostat alone, and MTX plus vorinostat. The MTX (50 mg/kg), vorinostat (50 mg/kg), or vehicle solutions were injected intraperitoneally at days 15, 18, and 21 from the subcutaneous injection. Vorinostat is relatively insoluble in aqueous solutions, and in previous mouse efficacy studies,^23^ it had been administered in 100% DMSO. Increased levels of histone acetylation resulting from intraperitoneal injection of 50 mg/kg vorinostat are readily detected in tumor tissue.^23^ Since 100% DMSO is not well tolerated on repeated injection, we used 2% DMSO + 30% PEG300 + 5% Tween80 + ddH_2_O as a vehicle for vorinostat. In the present study, intraperitoneal injection of 50 mg/kg vorinostat was tolerant (Supplementary Figure S4).

The tumor volumes were measured 3 times per week, which revealed significantly smaller tumors in the groups that received MTX alone (*P* < .0005) and MTX plus vorinostat (*P* < .0001). In addition, the combination of MTX plus vorinostat was significantly more effective than MTX alone (*P* < .0001; Figure 4B).

Based on those results, we also evaluated the combination of MTX plus vorinostat in the intracranial xenograft mice model. The TK cells were injected into the right cerebral hemispheres of 6-week-old male BALB/c-nu/nu mice (Figure 4C), which were then divided into 4 groups: vehicle control, MTX alone, vorinostat alone, and MTX plus vorinostat. The MTX (50 mg/kg), vorinostat (50 mg/kg), or vehicle solutions were injected intraperitoneally at days 4, 6, 8, and 10 from the intracranial injection. In the group treated with MTX alone, 3 of 11 mice died from the drug toxicity and 1 mouse failed to engraft. In the group treated with vorinostat alone, 3 of 11 mice failed to engraft. In the group treated with a combination of MTX plus vorinostat, 1 of 11 mice died from the drug toxicity. Mice that died from drug toxicity or failed to engraft were excluded from the survival analysis.

Based on survival, the combination of MTX plus vorinostat was significantly more effective than the vehicle control (*P* < .01) or MTX alone (*P* < .05). However, MTX alone or vorinostat alone was not significantly more effective than the vehicle control (Figure 4D). These results suggest that vorinostat enhanced the therapeutic effect of MTX thorough the long-lasting retention of MTX-PGs in the tumor cells.

## Discussion

Patients with PCNSL have varying responses to HD-MTX treatment with LV rescue. We have previously reported that the extent of polyglutamylation in tumor cells is responsible for treatment resistance. Moreover, we demonstrated that LV rescue after MTX treatment was significantly enhanced in cells with reduced polyglutamylation levels caused by FPGS knockdown (vs a scrambled-sequence control), while induction of MTX polyglutamylation using HDACI might be a more effective therapy for PCNSL.^16^

The present study revealed that the responses of lymphoma cell lines to MTX treatment with LV rescue were dependent on the FPGS/GGH ratio, which reflected the extent of MTX polyglutamylation. Furthermore, 4 kinds of HDACIs upregulated the FPGS/GGH ratio and downregulated DHFR expression, with vorinostat having the lower IC_50_ values in all cell lines. Finally, vorinostat has been reported to cross the blood–brain barrier, which prompted us to examine whether it would be effective when combined with MTX treatment. To the best of our knowledge, this is the first study to indicate that vorinostat increased the levels of long-chain MTX-PGs in lymphoma cells based on MS imaging, and that the combination of MTX plus vorinostat was more effective than MTX alone based on in vitro and in vivo experiments.

The mechanisms of MTX resistance involve a failure to accumulate MTX-PGs mainly due to decreased FPGS-catalyzed polyglutamylation, decreased MTX cell entry due to impaired membrane transport, or increased DHFR activity.^11,24–26^ To decrease MTX resistance in PCNSL treatment, induction of MTX polyglutamylation or decrease of DHFR activity by combinational therapy with MTX should be effective.

Galpin et al.^27^ identified a significantly lower cellular accumulation of MTX-PGs in T-lineage versus B-lineage lymphoblasts in children with acute lymphoid leukemia, which is consistent with the worse prognosis of T-lineage acute lymphoid leukemia when treated with conventional MTX-based therapy. They also showed that higher FPGS and lower DHFR levels are potential mechanisms contributing to greater MTX-PG accumulation and cytotoxicity in B-lineage lymphoblast cell lines than T-lineage lymphoblast cell lines in in vitro experiments.

In the present study, we evaluated the correlations of the MTX cytotoxic effect with the extent of polyglutamylation (FPGS/GGH) and DHFR expression level. The IC_50_ values for MTX were highest in HKBML and almost the same in TL-1 and TK cells. However, over 90% cytotoxicity was obtained only in TK cells even at the maximal cytotoxic dose of MTX. Compared with the difference between HKBML and TK cells, the extent of polyglutamylation was higher in TK cells, although the expression levels of DHFR were almost the same. These results were consistent with the lower IC_50_ value of MTX and over 90% cytotoxic effect obtained at the maximal effect in TK cells. Compared with the difference between TL-1 and TK cells, the degree of polyglutamylation was higher and the expression level of DHFR was lower in TK cells. These results indicated that MTX would be more effective against TK cells than TL-1 cells. Although the IC_50_ value of MTX was almost the same in these 2 cell lines, the maximal cytotoxic effects were higher in TK cells. These results were consistent with the results of the extent of polyglutamylation and DHFR expression level. The response to MTX treatment and LV rescue is associated with the extent of polyglutamylation. EC_50_ values for LV rescue were lowest in HKBML cells and highest in TK cells, which was consistent with the FPGS/GGH ratio, which nearly reflected the extent of MTX polyglutamylation.

HDACIs can upregulate global histone acetylation levels. The changes in histone acetylation levels at gene promoters tend to correlate with the changes in gene expression levels. HDACIs have been reported to exert multiple antitumor mechanisms, such as activation of differentiation, arrest of cell-cycle progression in G_1_ and/or G_2_, and apoptotic induction.^28–31^

Leclerc et al.^32^ demonstrated that Sp1 and NFY transcription factors bind strongly to the native chromatin structure of the FPGS promoter region, and these transcription factors cooperatively recruit HDAC1 to the FPGS promoter region; assembly of this complex would regulate FPGS mRNA expression epigenetically through chromatin remodeling. Consequently, treatment with HDACIs would alter the FPGS promoter acetylation status and increase FPGS gene transcription and intracellular accumulation of long-chain MTX-PGs based on in vitro tests of acute lymphoid leukemia cell lines.^32^

Vorinostat (molecular weight: 264) is a small-molecule inhibitor of most human class I and class II HDACs. The biological effects of HDAC inhibition via vorinostat are widespread, with HDAC inhibitors causing changes in 2–10% of all expressed genes.^33^ There is preclinical evidence that vorinostat has antitumor activity against malignant glioma cell lines in vitro and orthotopic xenografts in vivo.^22,34,35^ A phase II trial of vorinostat in patients with recurrent GBM20 also revealed that vorinostat was well tolerated, and paired tumor samples from baseline and post-vorinostat treatment revealed increased histone acetylation that was consistent with the expected targeted effect. Other findings have indicated that vorinostat crosses the blood–brain barrier.^20–22^ In the present study, induction of FPGS expression and the concomitant decrease in DHFR expression by vorinostat would enhance the synergism of the MTX and vorinostat combination in lymphoma cell lines. It was not clear whether the downregulation of DHFR expression resulted from the cell-cycle arrest induced by HDACIs or from a direct effect on gene expression.

Although vorinostat had a strong cytotoxic effect on lymphoma cell lines in in vitro experiments, single-agent administration of vorinostat was not effective in both subcutaneous and intracranial mouse models. Although there could be several mechanisms involved in the discrepancy, increased FPGS activity induced by vorinostat might be one possible mechanism: increased FPGS activity converts intracellular folates to polyglutamylated folates when antifolates are not administered together. Polyglutamylated folates facilitate DNA synthesis and cell proliferation. It has been reported that cells overexpressing FPGS grow faster than those expressing endogenous FPGS.^36^ Unlike in vitro experiments, there are abundant circulating folates in vivo from which vorinostat may induce polyglutamylated folates through the increased FPGS in the absence of antifolates such as MTX, which may result in lower direct cytotoxicity of vorinostat.

To evaluate the therapeutic effect of inducing MTX polyglutamylation, we examined the combination of MTX plus vorinostat in vivo. In this context, the serum and cerebrospinal fluid (CSF) concentrations of MTX are linked to dose escalation,^37^ as an MTX dose of 500 mg/m^2^ does not produce a potentially cytotoxic CSF concentration. However, a high dose of MTX (2500 mg/m^2^) was sufficient to achieve a cytotoxic CSF concentration in humans. Our preliminary data have indicated that the maximum tolerable dose of MTX was 50 mg/kg for mice, which avoids severe adverse events in many mice (data not shown), and this is the dose we selected for the present study. However, we failed to detect a therapeutic effect for MTX alone in the intracranial xenograft mouse model, which suggests that this dose may not have been effective as shown in Figure 4C. Furthermore, based on a practice guide for dose conversion between animals and humans,^38^ the dose of 50 mg/kg for mice would convert to approximately 150 mg/m^2^ in humans. Thus, the dose of MTX in the intracranial xenograft model was too low for a sufficient amount of MTX to pass through the blood–brain barrier. However, increased polyglutamylation and decreased DHFR expression by the administration of vorinostat might enhance the cytotoxicity of MTX, even if the concentration of MTX does not reach a high-dose level.

We evaluated the polyglutamylation of MTX in cells indirectly by the ratio of FPGS/GGH and directly using MALDI-MS imaging. However, we could not detect the polyglutamylation of MTX in specimens of the intracranial xenograft model, possibly due to technical difficulty, which is a future issue to be solved.

In conclusion, our results revealed that the pharmacological advantage of the combination of MTX plus HDACIs in lymphoma cells stemmed from their synergistic effects on the increased synthesis of long-chain MTX-PGs and decreased expression of DHFR by HDACIs, which is a main target of MTX. The combination of HD-MTX-based chemotherapy plus vorinostat might be effective for treating PCNSL.

## Supplementary Material

vdaa084_suppl_Supplementary_Figure_S1

vdaa084_suppl_Supplementary_Figure_S2

vdaa084_suppl_Supplementary_Figure_S3

vdaa084_suppl_Supplementary_Figure_S4

vdaa084_suppl_Supplementary_Figure_S5

vdaa084_suppl_Supplementary_Fig_Legend_and_Methods

## Notes

**Authorship statement.** Study conception and design: K.F. and N.S.; data acquisition: K.F., M.H., and T.N; data analysis and interpretation: K.F., M.H., N.S., K.I., and A.M; drafting of the manuscript: K.F.; review of the manuscript: N.S.; critical revision: K.I. and A.M.
