# Supplementary figures and images for: Histone deacetylase inhibition enhances the therapeutic effects of methotrexate on primary central nervous system lymphoma
Source: Neurooncol Adv. 2020 Jul 3;2(1):vdaa084. doi: 10.1093/noajnl/vdaa084 (PMC7415262; doi:10.1093/noajnl/vdaa084)

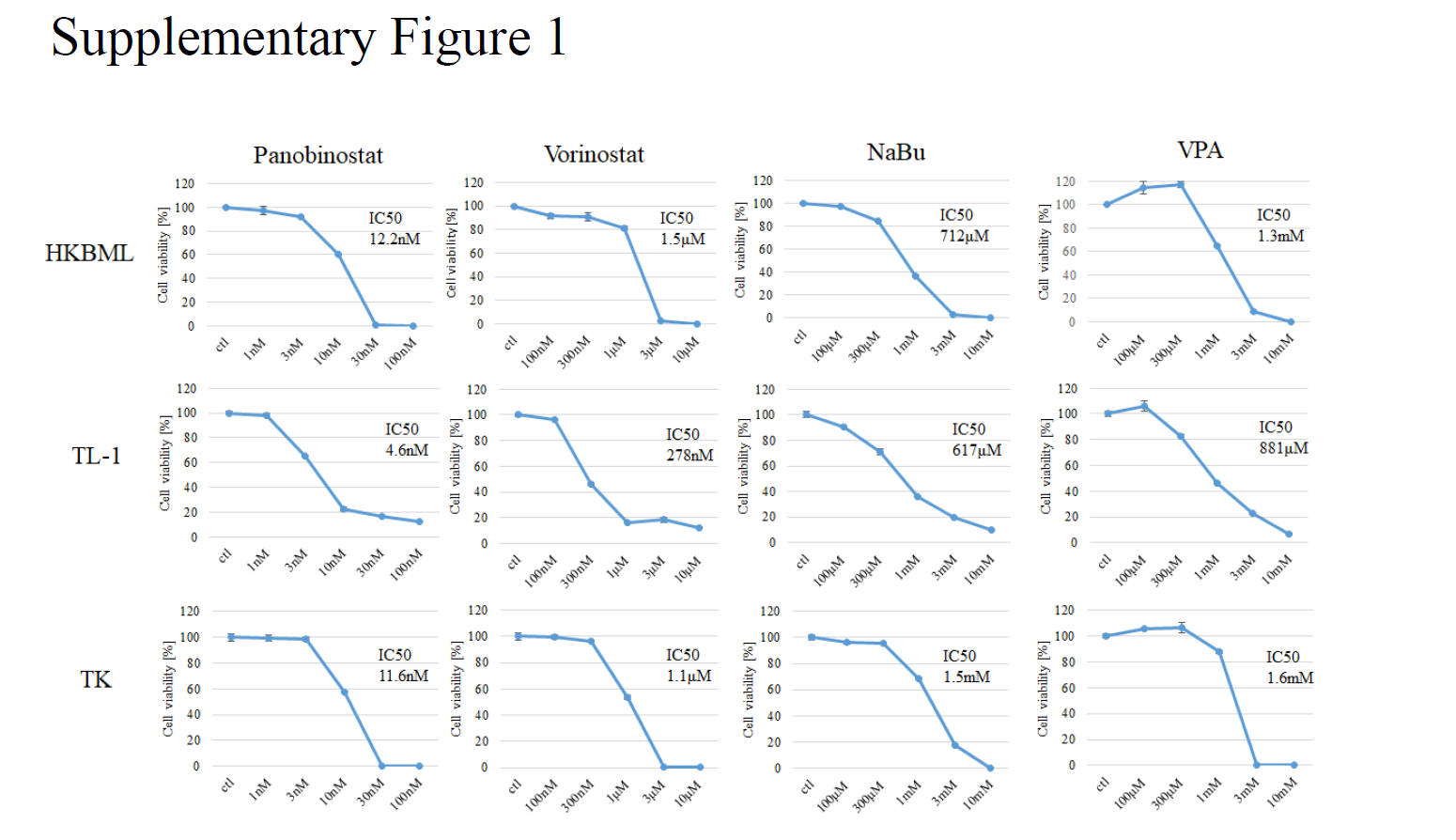

Supplement: vdaa084_suppl_Supplementary_Figure_S1 [file vdaa084_suppl_supplementary_figure_s1.png]

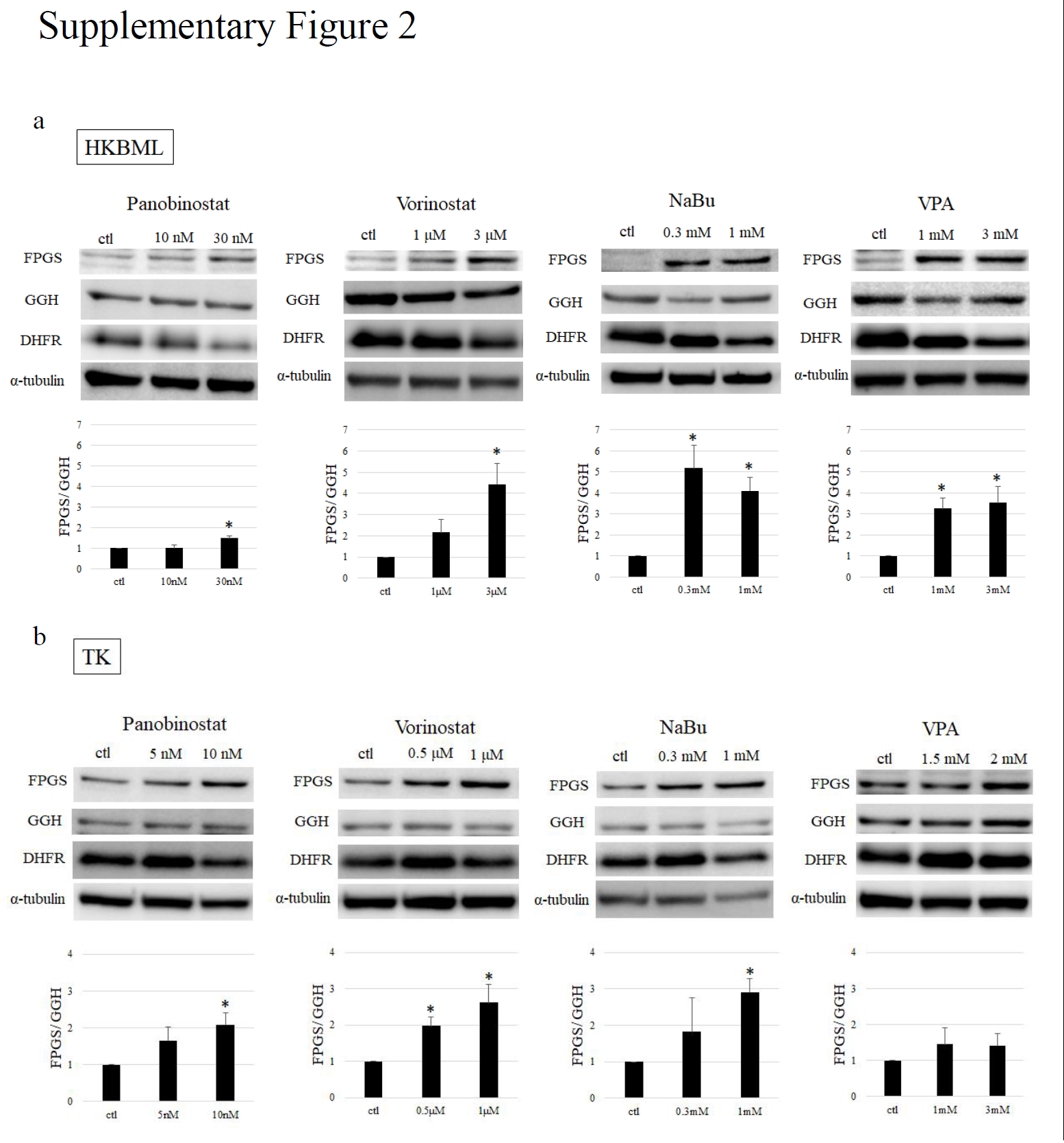

Supplement: vdaa084_suppl_Supplementary_Figure_S2 [file vdaa084_suppl_supplementary_figure_s2.png]

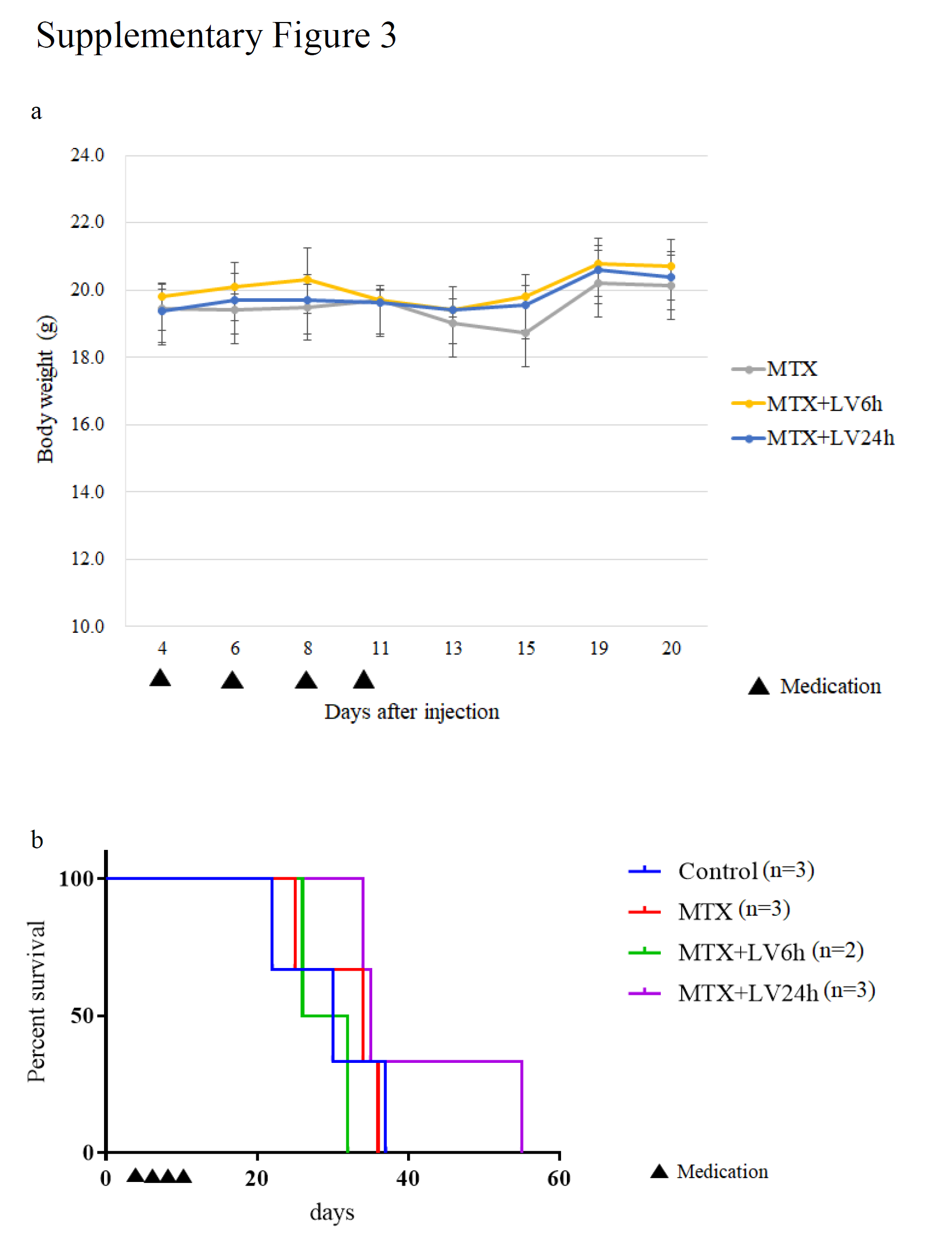

Supplement: vdaa084_suppl_Supplementary_Figure_S3 [file vdaa084_suppl_supplementary_figure_s3.png]

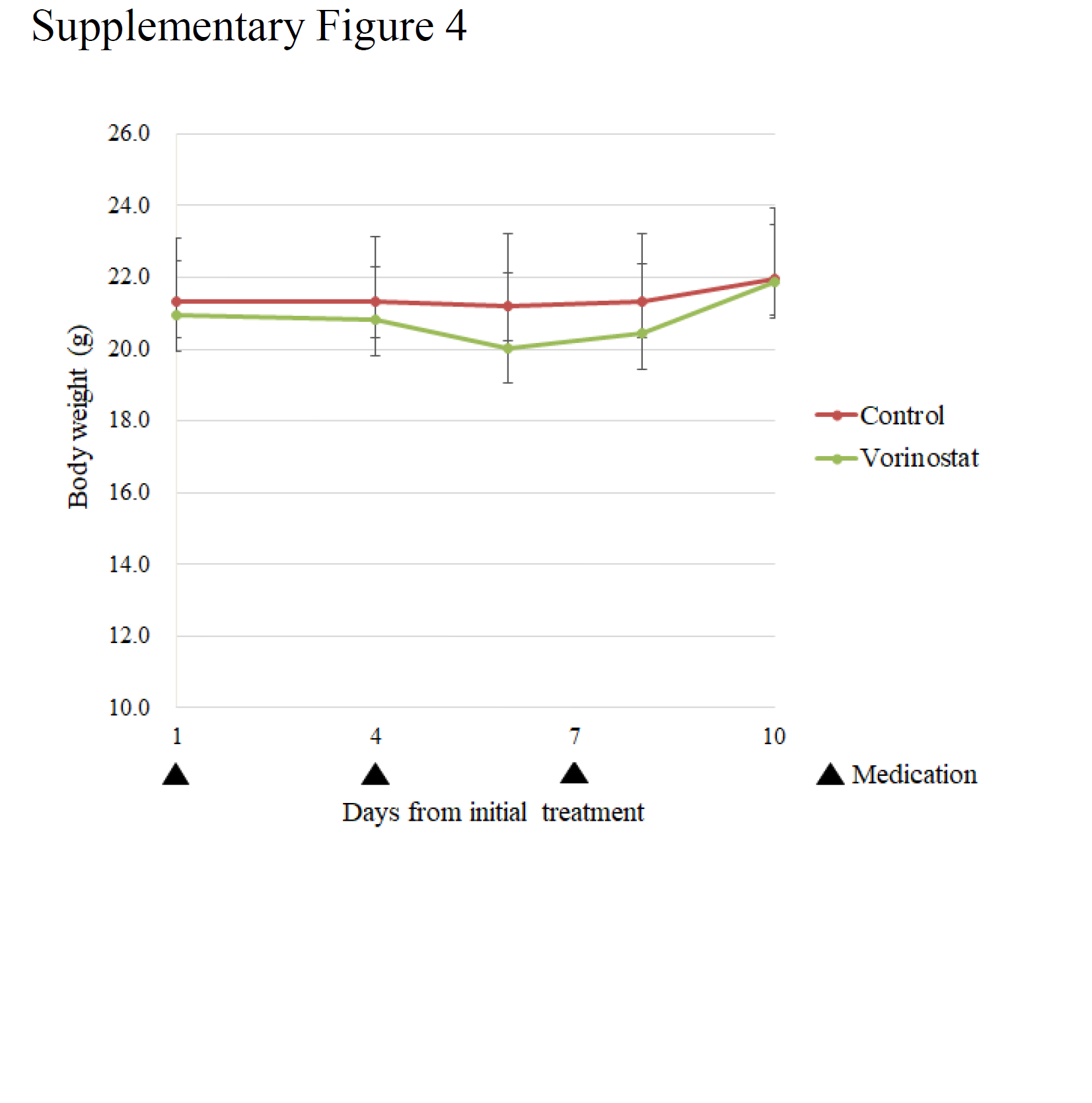

Supplement: vdaa084_suppl_Supplementary_Figure_S4 [file vdaa084_suppl_supplementary_figure_s4.png]

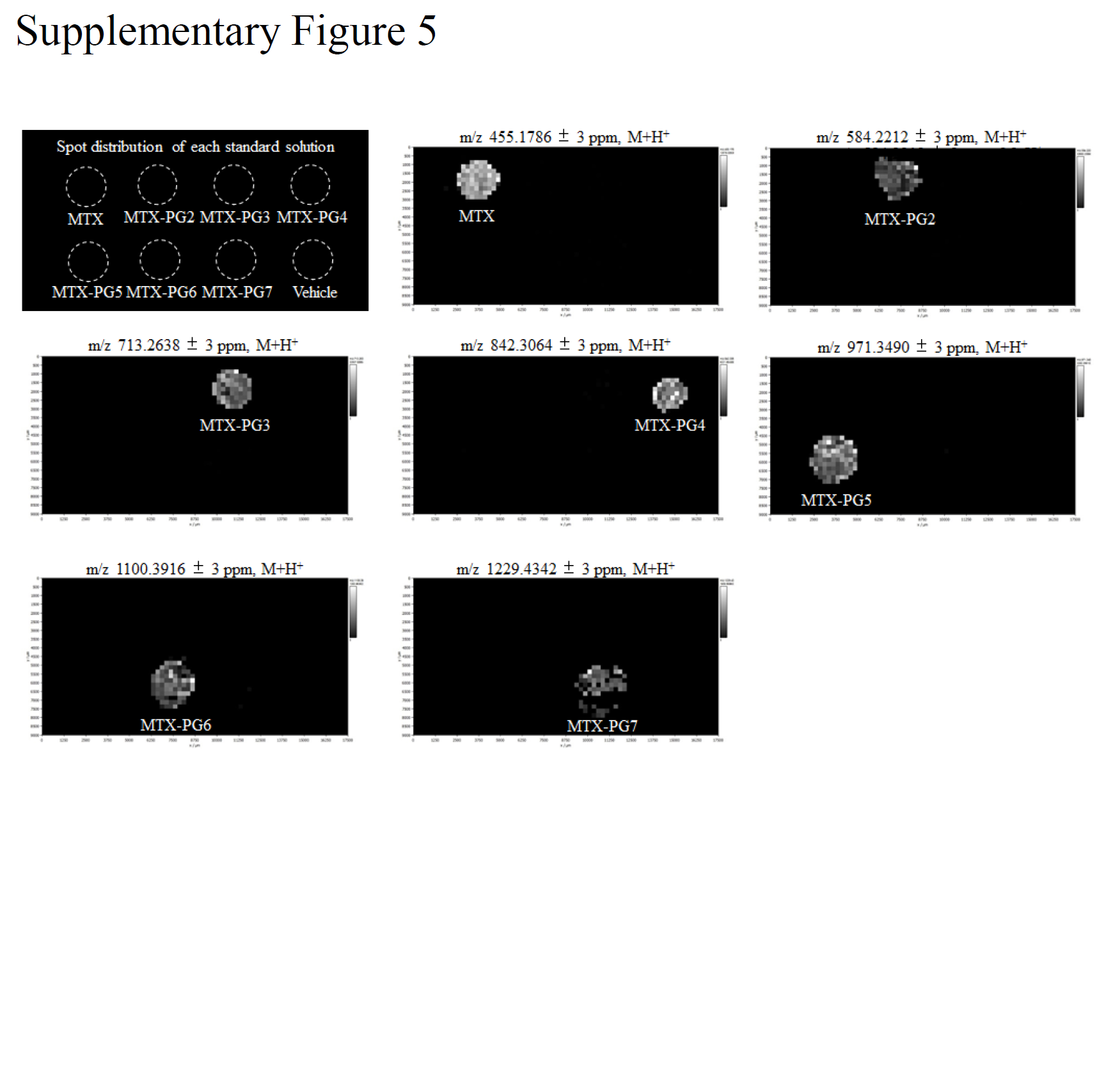

Supplement: vdaa084_suppl_Supplementary_Figure_S5 [file vdaa084_suppl_supplementary_figure_s5.png]
