# Supplementary material for: Histone deacetylase inhibition enhances the therapeutic effects of methotrexate on primary central nervous system lymphoma
Source: Neurooncol Adv. 2020 Jul 3;2(1):vdaa084. doi: 10.1093/noajnl/vdaa084 (PMC7415262; doi:10.1093/noajnl/vdaa084)
Supplement: vdaa084_suppl_Supplementary_Fig_Legend_and_Methods [file vdaa084_suppl_supplementary_fig_legend_and_methods.docx]

**Supplementary figure legends**

Figure S1: Effects of panobinostat, vorinostat, sodium butyrate (NaBu), and valproic acid (VPA) on lymphoma cell lines. Cell viability was examined after 72 h of incubation with each drug (CellTiter-Glo).

Figure S2: Changes in expression of folypolyglutamate synthetase (FPGS), γ-glutamyl hydrolase (GGH), and dihydrofolate reductase (DHFR) after treatment using histone deacetylase inhibitors (HDACIs) for 72 h. (a) HKBML cells and (b) TK cells. The relative expression of FPGS/GGH represents the ratio of FPGS/α-tubulin and GGH/α-tubulin calculated by densitometry. The FPGS/ GGH ratio of HKBML or TK control is adjusted to 1. Data are shown as mean value ± standard deviation from 3 independent experiments. *P<0.05

Figure S3: Effects of methotrexate (MTX) treatment and leucovorin (LV) rescue in an intracranial xenograft model. (a) Body weight changes over time (n=3, respectively) and (b) Kaplan-Meier survival curves for each treatment group: control (n=3), MTX (n=3), MTX+LV at 6 h (n=2), and MTX+LV at 24 h (n=3). MTX and LV are used at a dose of 50 mg/kg. In the group of MTX + LV rescue at 6 h, one mouse failed to engraft, so the mouse was excluded from the survival analysis.

Figure S4:

Body weight changes over time of mice treated with vorinostat (50 mg/kg) or a control (n=5 each). Error bars indicate the standard deviation.

Figure S5: Matrix-assisted laser desorption/ionization mass spectrometry imaging of the spot distributions for methotrexate (MTX) and the MTX-PG2–7 standards.

**Supplementary Methods**

*Matrix-assisted laser desorption/ionization mass spectrometry imaging (MALDI MS imaging)*

The frozen cell pellet was rapidly adhered to the glass slide (PRO-11, Matsunami Glass) using warmth from the researcher’s fingers. The cells were coated with α-cyano-4-hydroxycinnamic acid (CHCA, #476870, Sigma-Aldrich) using the matrix preparation robot (SMALDI-Prep, TransMIT) according to the manufacturer’s protocol. The MS data were acquired using an ion source for high-resolution atmospheric pressure MALDI MS Imaging (AP-SMALDI-10, TransMIT) and a hybrid quadrupole-orbitrap mass spectrometer (Q Exactive, Thermo Fisher Scientific). The optimized parameters were: spatial resolution, 15 μm; laser attenuator, 15 and 10% filter on; mass resolution, 140,000 at m/z 200; AGC target, 1e6; spray voltage, 4.3 kV; capillary temperature, 250°C; and S-lens level, 80. The scan scenario was s full scan for m/z 450–1,300 in cation mode. Molecular data and image processing were performed using Mirion software (v3.2.64.18, TransMIT), and methotrexate (MTX) matches were identified based on a matching accurate mass with 3 ppm tolerance: MTX [M+H]^+^ (m/z 455.1786), MTX-PG2 [M+H]^+^ (m/z 584.2212), MTX-PG3 [M+H]^+^ (m/z 713.2638), MTX-PG4 [M+H]^+^ (m/z 842.3064), MTX-PG5 [M+H]^+^ (m/z 971.3490), MTX-PG6 [M+H]^+^ (m/z 1100.3916), MTX-PG7 [M+H]^+^ (m/z 1229.4342), and PC(34:1) [M+K]+ (m/z 798.5410). Peaks in a non-cell region were used as a reference for background noise (Fig. S5). Relative amounts of MTX polyglutamylations were calculated by correcting the numbers of each detected pixel with phospholipid PC (34:1).

*In vivo evaluation of MTX treatment and LV rescue in an intracranial xenograft model*

For the *in vivo* evaluation of MTX treatment and LV rescue, we used an intracranial tumor-bearing BALB/c-nu/nu mouse xenograft model (Charles River, Yokohama, Japan). The TK cells (2.0 × 10^5^ cells in 2 μL of PBS) were injected into the right cerebral hemispheres of 6-week-old male BALB/c-nu/nu mice using a Hamilton syringe and a stereotactic micro-injector (Narishige, Tokyo, Japan). Four days later, the mice were randomly divided into 4 groups: control, MTX alone, and MTX treatment plus LV rescue at 6 h (MTX+LV6h) or at 24 h (MTX+LV24h). The control vehicle (0.05M Na_2_CO_3_ diluted to 50% with saline) or MTX (50 mg/kg) were injected intraperitoneally at days 4, 6, 8, and 10. Leucovorin (50 mg/kg) was injected intraperitoneally at 6 or 24 h after MTX treatment. Body weight and survival time were monitored and analyzed using the Kaplan-Meier method. All animal experiments were approved by the Animal Experimental Committee of the National Cancer Center and were performed in accordance with the Guidelines for Animal Experiments of the National Cancer Center, which concur with the ethical guidelines for animal experiments in Japan.
